# Dual inhibition of thrombin and activated factor X attenuates disseminated intravascular coagulation and protects organ function in a baboon model of severe Gram-negative sepsis

**DOI:** 10.1186/s13054-017-1636-y

**Published:** 2017-03-13

**Authors:** Herbert Schöchl, Martijn van Griensven, Stefan Heitmeier, Volker Laux, Ulrike Kipman, Jan Roodt, Soheyl Bahrami, Heinz Redl

**Affiliations:** 1 Ludwig Boltzmann Institute for Experimental and Clinical Traumatology, AUVA Research Centre, Vienna, Austria; 2 Department of Anesthesiology and Intensive Care Medicine, AUVA Trauma Centre Salzburg, Academic Teaching Hospital of the Paracelsus Medical University, Dr. Franz Rehrl Platz 5, 5020 Salzburg, Austria; 3 Department of Experimental Trauma Surgery, Trauma Surgery, Klinikum rechts der Isar, Technical University of Munich, Munich, Germany; 4 Bayer Pharma AG, Acute Care Research, Wuppertal, Germany; 5 UT SPSS Statistics, Salzburg, Austria; 6 Department of Haematology and Cell Biology, Faculty of Health Sciences, University of the Free State, Bloemfontein, Free State South Africa

**Keywords:** DIC, Inflammation, MOF, Short-acting coagulation factor II/Xa inhibitor, SATI

## Abstract

**Background:**

Inhibition of procoagulant pathways may improve outcome in sepsis. We examined whether a dual short-acting thrombin (factor II) and factor X (FX)a inhibitor (SATI) ameliorates sepsis-induced disseminated intravascular coagulation (DIC) and is organ-protective.

**Methods:**

*Escherichia coli* were infused for 2 h in 22 anesthetized baboons. The control (CO) group (n = 8) received sterile isotonic solution only. In the treatment groups, SATI was administered starting 15 minutes after the end of the bacterial exposure. In the low-dose group (LD-SATI, n = 8), SATI was infused with 75 μg/kg/h for the first hour, followed by 23 μg/kg/h until the end of the study. In the high-dose SATI group (HD-SATI, n = 6), 225 μg/kg/h was administered for the first hour followed by continuous infusion of 69 μg/kg/h until termination of the study.

**Results:**

Sepsis-induced DIC was attenuated, as reflected by lower peak thrombin-antithrombin complexes (threefold) and D-dimer levels (twofold) in both SATI groups compared to the CO. This coincided with strongly improved cell/organ protection assessed by decreased levels of lactate dehydrogenase (threefold), creatinine (twofold), aspartate aminotransferase (threefold), and amylase (twofold) compared to the CO group. Anuria, which started at 8 h in the CO group, was prevented in both SATI groups. Peak interleukin-6 release at 12 h was prevented in the treatment groups. In both SATI groups, fewer catecholamines were necessary and no bleeding complications were observed.

**Conclusions:**

Dual inhibition of thrombin and FXa preserved activation of coagulation, protected organ function and ameliorated inflammation in severe Gram-negative sepsis in baboons. SATI could be a novel therapeutic agent against sepsis-induced DIC.

## Background

Severe sepsis and septic shock remain a prominent cause of mortality in hospital, exceeding 30% [1, 2]. Bacterial invasion not only prompts a potent systemic inflammatory response, but it also results in compelling upregulation of the coagulation system [3–7]. Thus, the majority of patients with severe sepsis suffer from coagulation disorders ranging from minor changes in activation markers to full-blown disseminated intravascular coagulation (DIC) [7, 8].

Experimental studies demonstrate that tissue factor (TF) expression, in particular on monocytes and endothelial cells play a key role in the pathogenesis of DIC [7]. TF complexes with factor VII (FVII) and subsequently activates factor X (FX) and factor IX (FIX) with a strong downstream effect on thrombin generation [9]. This procoagulant stimulus in alliance with sustained depression of fibrinolytic pathways results in disseminated intravascular formation of microthrombi [5]. Widespread fibrin deposition in the microcirculation of various organs is closely linked to the development of multiple organ failure and has been identified as an important contributor of morbidity and mortality [5, 10]. For example, Dhainaut et al. reported that mortality increased from 27% in patients with sepsis without DIC to 43% in those with accompanying DIC [11].

Restoration of anticoagulant pathways by administration of antithrombin (AT) III, recombinant activated protein C (APC) and recombinant tissue factor pathway inhibitor (TFPI) in patients with sepsis without DIC has not resulted in survival benefits in randomized controlled trials [12–16]. In contrast, ATIII and recombinant APC in patients with overt DIC showed promising results towards improved outcome [17–19].

Direct thrombin inhibitors such as lepirudin and the predominant factor Xa inhibitor Danaparoid have been investigated in endotoxin models. Both substances are sufficient to hinder activation of coagulation [20, 21]. Until now, no studies investigated a combined thrombin and FXa inhibitor in severe sepsis. Dual inhibition of thrombin and FXa in part mimics the natural anticoagulant ATIII, which effectively inhibits both enzymes [22]. While ATIII binds thrombin in a 1:1 ratio, direct thrombin and FXa inhibitors block the active side of the corresponding coagulation factors and are not dependent on sufficient levels of cofactors such as heparin [23].

In the current study, we examined the efficacy and safety of a new potent, short-acting, reversible and selective combined FIIa and FXa inhibitor (short-acting thrombosis inhibitor (SATI)) in an established experimental model of severe *Escherichia coli* sepsis in baboons [24]. The hypothesis of the current investigation was that the administration of SATI in severe sepsis will attenuate activation of coagulation (with consecutive microthrombi formation), diminish DIC and preserve organ function.

## Methods

The experimental protocol was approved by the Institutional Animal Care use Committee at Free State University Bloemfontein, South Africa (number 03/2010). All experiments were performed under the conditions described in the Guide for the Care and Use of Laboratory Animals as defined by the National Institutes of Health.

Twenty-two healthy male Chacma baboons of the strain *Papio ursinus*, with a median weight of 18.1 (16.5–22.3) kg and aged between 3 and 10 years were included in the current study. The animals were quarantined for 3 months prior to the study. The primates were fasted overnight before experiments, with free access to water.

### Premedication, anesthesia and instrumentation of the animals

Premedication of the baboons was performed by intramuscular injection of 6–8 mg/kg body weight (BW) ketamine hydrochloride (Ketalar®, Pfizer, Vienna, Austria). After placing the animal in the supine position, the right cubital vein was cannulated and anesthesia was induced using 5 mg/kg BW sodium pentobarbital (Sandoz GmbH, Kundl, Austria) and maintained by continuous infusion of 0.8 mg/kg/h sodium pentobarbital, 0.8 μg/kg/h, sufentanil (Janssen, Vienna, Austria), and 1 mg/kg/h rocuronium (Organon, Oss, Netherlands).

Following endotracheal intubation the animals were ventilated in volume-controlled mode using a tidal volume of 6–8 ml/kg and a respiratory rate of 18–20 breaths/minute in order to maintain an arterial pressure of CO_2_ at 35–45 mmHg (Evita 2, Dräger, Lübeck, Germany). The fraction of inspired oxygen was set at 0.30 and adjusted if necessary according to blood gas analyses. Body temperature was kept at 37 °C.

Three catheters were placed using the Seldinger technique: (1) for blood pressure monitoring and blood sampling a catheter was inserted into the right femoral artery; (2) the cephalic vein was used for fluid therapy and administration of SATI; and (3) a Swan-Ganz catheter (Edwards Lifesciences, Irvine, CA, USA) was inserted via the femoral vein for measurement of central venous pressure (CVP), pulmonary arterial pressure and, pulmonary artery occlusion pressure (PAOP), and hemodynamic variables such as cardiac index (CI), systemic vascular resistance index (SVRI) and pulmonary vascular resistance index (PVRI). A transurethral catheter was placed to quantify urine output at baseline, and at 2-h intervals for 8 h, followed by 4-h intervals for 24 h).

### Experimental design

**Fig. 1:**
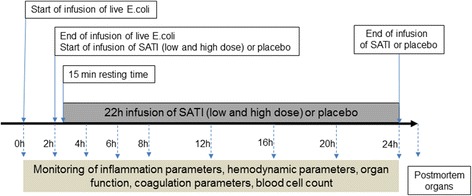
Study protocol. *SATI* short-acting thrombosis inhibitor

After a stabilization period, 5 × 10^6^ colony-forming units (CFU)/kg *E. coli* O111:K58(B4):H-(ATCC 33780) were infused under continuous stirring over a 2-h period via a motor-pump as previously described [25] (Fig. 1). Ringer’s solution (Fresenius-Kabi GmBH, Bad Homburg, Germany) of at least 5 ml/kg/h was infused to maintain mean arterial blood pressure (MAP) at >70 mmHg and CVP and PAOP at >5 mmHg. If these target values were not established, higher rates of Ringer’s solution or noradrenalin (or if necessary adrenalin) were administered. Antibiotics were not used.

To investigate the optimal dose for efficacy and safety, two treatment regimens were evaluated: for the lower dose a plasma concentration was selected that led to a prothrombin time (PT) prolongation of 1.4 in healthy animals. This concentration was chosen, because it resulted in a significant reduction in thrombus weight of about 50% in an arteriovenous shunt thrombosis model in rats, which has previously been used for predictions of concentration in the setting of acute thrombosis. As expected, a threefold higher concentration led to approximately 80% reduction in thrombus weight, but it did not lead to a significant increase in bleeding time. Thus, these two doses were chosen to investigate whether the lower dose is as efficacious as in acute arterial thrombosis scenarios and whether the higher dose would have even more efficacy with an acceptable bleeding profile.

At 15 minutes after completion of the bacterial infusion the animals were randomly assigned to one of three groups. The control (CO) group (n = 8) received a crystalloid solution as vehicle (Ionosteril, Fresenius-Kabi GmBH, Bad Homburg, Germany). The treatment groups received either low-dose SATI (LD-SATI) or high-dose SATI (HD-SATI). The LD-SATI group (n = 8) started with 75 μg/kg/h for the first hour followed by 23 μg/kg/h until the end of the study. In the HD-SATI group (n = 6), 225 μg/kg/h was administered for the first hour followed by continuous infusion of 69 μg/kg/h until the end of the study. SATI was dissolved in 50 ml of vehicle. At 24 h after the start of the bacterial infusion, the animals were killed by an intravenous (i.v.) bolus injection of 100 mg/kg pentobarbital followed by 10 ml (1 mmol/ml) potassium chloride.

### Blood sampling

Baseline blood samples for blood gas analysis, blood cell count and clinical chemistry analysis were obtained after 1 h, 2 h and then at every 2 h within the first 8 h, followed by sampling every 4 h until the end of the experiment (24 h) (Fig. 1). Blood cell counts were determined by a Coulter T890 counter (Coulter Electronics Inc., Hialeah, FL, USA). Coagulation analyses were performed in the blood collected in 3-ml tubes containing 0.3 ml buffered 3.2% trisodium citrate, giving a volume ratio of 1 + 9 (Vacuette; Greiner Bio-One, Linz, Austria). Blood was centrifuged immediately at 2000 *g* for 10 minutes to obtain plasma, which was stored at –80 °C until analysis.

### Coagulation tests

The coagulation tests were performed according to the manufacturer’s instructions: antithrombin (AT) (AssayMax human Antithrombin III ELISA; AssayPro, St. Charles, MO, USA); D-dimer (Asserachrom D-Dimer ELISA, Diagnostica Stago, Taverny, France); fibrinogen (Human Fibrinogen ELISA, ICL, Portland, USA); PAI-1 antigen (Technozym ELISA, Cryopep, France); activated protein C (human activated Protein C ELISA, Wuhan USCNK, Houston, USA); thrombin-antithrombin-complexes (Enzygnost TAT micro, ELISA, Siemens, Marburg, Germany); tissue-plasminogen-activator activity (Technozym ELISA t-PA, Technoclone, Vienna, Austria); tissue factor pathway inhibitor (Quantikine human TFPI ELISA, R&D Systems, Minneapolis, USA), thrombin activated fibrinolysis inhibitor (Zymotest TAFI Antigen; Hyphen, Neuville-sur-Oise, France) and thrombomodulin (Diagnostica Staro, Taverny, France).

### Inflammation markers

Lipopolysaccharide binding protein (LBP), interleukin (IL)-6, and IL-10 were determined using ELISA assays and performed on Immulite® 1000 (Siemens Healthcare, Erlangen, Germany). Tumor necrosis factor-α (TNF-α) was measured by ELISA kits (Bender MedSystems, Vienna, Austria) according to the manufacturer’s instructions.

### Blood gases and chemistry

Arterial and venous blood gases were measured (Radiometer ABL 330, Copenhagen, Denmark). Renal (urea, creatinine, uric acid), liver (aspartate-Aminotransferase (AST), alanine-aminotransferase (ALT) and bilirubin) and pancreas parameters (amylase, lipase) and cell damage parameters (lactate dehydrogenase (LDH) were analyzed using the Sysmex XE-2100 (Sysmex, Kobe, Japan).

### Statistical analyses

To investigate group differences and changes in organ function parameters due to the intervention in an overall analysis, we performed repeated measures analysis of variance (ANOVA) with time as a within-subjects-factor and group as a between-subjects-factor for each parameter. The start of SATI administration was chosen as the reference time point for the comparisons between the CO and treatment groups. Post hoc analyses were conducted using the Scheffé test. Analysis of differences was performed separately for each time point using the *t* test for independent samples (Bonferroni-corrected). For categorical variables, the chi square test was used. *P* values <0.05 were considered statistically significant. All statistical calculations were performed using commercially available statistical software (SPSS 23.0) and figures were created using GraphPad Prism 5.03. Data are expressed as mean and standard deviation (SD) or median and interquartile range.

## Results

Of the 22 baboons, 20 survived until the end of the experiment; the two deaths were encountered in the LD-SATI group. Importantly, no severe and/or life-threating bleeding complications (e.g. mucosal bleeding, bleeding from catheter insertion sites or hematuria) were observed either in the CO group or in either of the SATI treatment groups.

### Sepsis-related coagulopathy

**Fig. 2:**
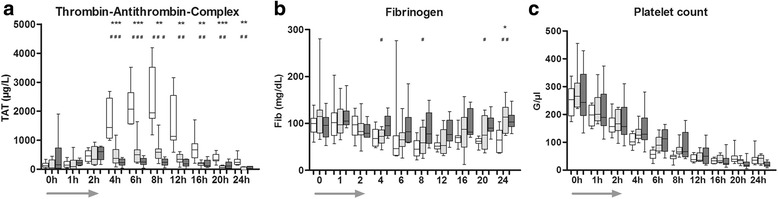
**a**-**c** Thrombin-antithrombin complexes (*TAT*), fibrinogen (Fib) concentration and platelet count starting from baseline (time point *0 h*) until the end of the experiment (time point *24 h*). *Arrow* represents the duration of *E. coli* infusion (first 2 h). *White* control group (CO), *light gray* low-dose short-acting thrombosis inhibitor (LD-SATI), *dark gray* high-dose (HD)-SATI. SATI prevented TAT formation and consumption of fibrinogen but the decrease in platelet count was unaffected. **a** TAT. **b** Fibrinogen CO vs LD-SATI ***p < 0.001, **p < 0.01, *p < 0.05; CO vs HD-SATI ^###^ *p* < 0.001, ^##^ *p* < 0.01, ^#^ *p* < 0.05; no indication = not significant. Two-factor analysis of variance was used for between-group comparisons. *Gray arrow* represents the time of the *E. coli* infusion

The bacterial challenge resulted in strong procoagulant activation indicated by high thrombin-anti-thrombin (TAT) complexes in the CO group. SATI administration abolished TAT complex formation (*p* < 0.0001) in the treatment groups compared to the CO group (peak, threefold higher), and significantly attenuated fibrinogen consumption in the HD-SATI group (HD-SATI vs the CO group, *p* < 0.02). All animals developed severe thrombocytopenia after infusion of *E. coli*, without any significant differences between the CO group and the two SATI groups (Fig. 2a-c).

**Fig. 3:**
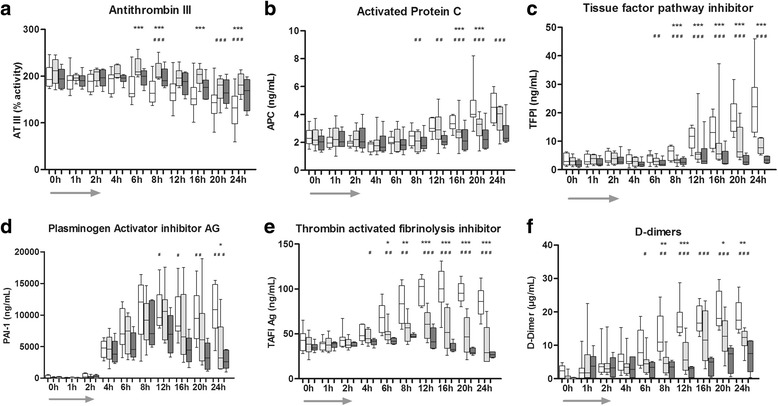
**a**-**f** Natural coagulation inhibitors, natural fibrinolysis inhibitors and D-Dimers starting from baseline (time point *0 h*) until the end of the experiment (time point *24 h*). *Arrow* represents the duration of *E. coli* infusion (first 2 h). W*hite* control group (CO), *light gray* low-dose short-acting thrombosis inhibitor (LD-SATI), *dark gray* high-dose (HD)-SATI. **a** Antithrombin III (*AT III*) ; **b** activated protein C (*APC*); **c** tissue factor pathway inhibitor (*TFPI*); **d** plasminogen activator inhibitor 1 (*PAI-1*); **e** thrombin-activated fibrinolysis inhibitor antigen (*TAFI*); **f** D-Dimer. CO vs HD-SATI ***p < 0.001, **p < 0.01, *p < 0.05; CO vs HD-SATI ^###^ *p* < 0.001, ^##^ *p* < 0.01, ^#^ *p* < 0.05; no indication = not significant. Two-factor analysis of variance was used for between-group comparisons. *Gray arrow* represents the time of *E. coli* infusion

ATIII significantly decreased over time in all groups (*p* < 0.0001), but only LD-SATI preserved ATIII consumption (*p* ≤ 0.02). APC increased significantly until the end of the experiment (*p* < 0.0001). HD-SATI administration resulted in lower APC values compared to the CO group (time point 8 h from baseline (TP 8), *p* < 0.014). Soluble Thrombomodulin (sTM) strongly increased over the observation period without significant differences between groups (data not shown). In the CO group, TFPI increased significantly until the end of the experiment (*p* < 0.0001). Animals in both treatment groups had lower TFPI concentrations compared to those in the CO group (both *p* < 0.0001). In particular, HD-SATI abolished upregulation of TFPI almost completely (Fig. 3a-c).

D-Dimers significantly increased over time in all groups reaching the highest level at TP 20 (*p* < 0.0001). Significant differences between the CO and LD-SATI groups (*p* ≤ 0.005) and the HD-SATI group (*p* ≤ 0.001) were detected. D-dimers at TP 12 were twofold higher in the CO compared to the treatment groups. HD-SATI appeared to be more efficient in attenuating D-Dimer production compared to LD-SATI. Plasminogen activator inhibitor 1 antigen (PAI-1) significantly increased over the observation period in all groups reaching the highest levels at TP 12. HD-SATI treatment resulted in significantly lower levels of PAI-1 compared to control (*p* = 0.03). Thrombin-activated fibrinolysis inhibitor antigen (TAFI) strongly increased in the CO group reaching the highest level at TP 16 (*p* < 0.0001). TAFI activation was sufficiently attenuated (*p* < 0.0001) in both SATI groups (Fig. 3d-f).

### Sepsis-induced organ damage

**Fig. 4:**
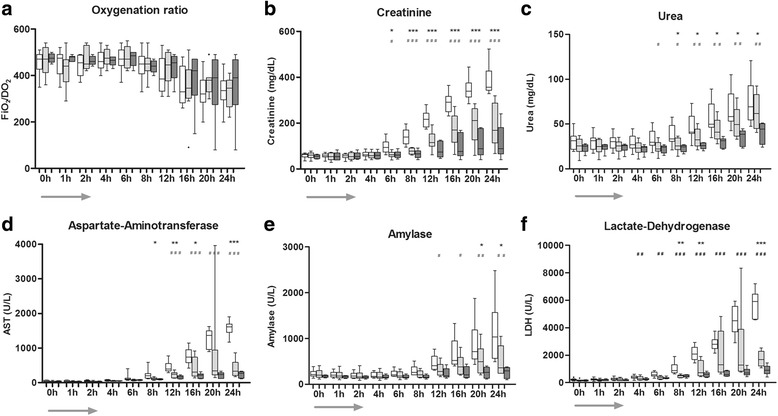
**a**-**f** Organ function starting from baseline (time point *0 h*) until the end of the experiment (time point *24 h*). *Arrow* represents the duration of *E. coli* infusion (first 2 h). *White* control group (CO), *light gray* low dose short-acting thrombosis inhibitor (LD-SATI), *dark gray* high-dose (HD)-SATI). *LDH* lactate dehydrogenase, *AST* aspartate aminotransferase. **a** Oxygenation ratio (*FiO2/paO2*); **b** creatinine; **c** urea; **d** aspartate-amino-transferase (*AST*); **e** amylase; **f** lactate-dehydrogenase (LDH). CO vs HD-SATI ***p < 0.001, **p < 0.01, *p < 0.05; CO vs HD-SATI ^###^ *p* < 0.001, ^##^ *p* < 0.01, ^#^ *p* < 0.05. Two-factor analysis of variance was used for between-group comparisons. *Gray arrow* represents the time of E. coli infusion

Oxygenation significantly deteriorated over the observation period in all groups as indicated by a steady decrease in the ratio of arterial oxygen partial pressure to fractional inspired oxygen (paO_2_/FiO_2_) ratio (*p* < 0.0001) (Fig. 4a) without significant differences between groups.

All animals in the CO group were anuric after TP 8, while urine production did not stop in any of the SATI-treated baboons. Both creatinine and urea significantly increased over time in all groups. However, creatinine was twofold higher in the CO group compared to both SATI treatment arms at TP 24 (*p* < 0.0001). Similarly, urea was twofold higher in the CO compared to the HD-SATI group (*p* ≤ 0.03) (Fig. 4b, c).

Treatment with LD-SATI and with HD-SATI lowered circulating AST threefold compared to CO at 24 h (*p* < 0.001). Amylase increased significantly in the course of the study (*p* < 0.005); SATI treatment (regardless of the dose) significantly abolished release of amylase (*p* < 0.05) compared to CO. LDH strongly increased over time in all groups (*p* < 0.0001). SATI treatment in both dose groups resulted in threefold lower LDH release compared to the CO (both *p* < 0.0001), (Fig. 4 d-f).

### Inflammation parameters after *E. coli* administration

**Fig. 5:**
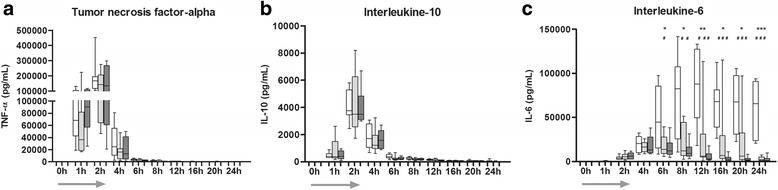
**a**-**f** Inflammatory cytokines starting from baseline (time point 0) until the end of the experiment (time point 24 h). *Arrow* represents the duration of *E. coli* infusion (first 2 h). *White* control group (CO), *light gray* low-dose short-acting thrombosis inhibitor (LD-SATI), *dark gray* high-dose (HD)-SATI. SATI prevented increase in interleukin 6 (*IL-6*) release in both treatment groups. CO vs LD-SATI group ***p < 0.001, **p < 0.01, *p < 0.05; CO vs HD-SATI ^# # #^ *p* < 0.001, ^# #^ *p* < 0.01, ^#^ *p* < 0.05; no indication = not significant. Two-factor analysis of variance was used for between-group comparisons. *Gray arrow* represents the time of *E. coli* infusion. *TNF-α* tumor necrosis factor alpha, *AT III* antithrombin III, *APC* active protein C, *TFPI* tissue factor pathway inhibitor, *TAFI* thrombin-activated fibrinolysis inhibitor antigen, *PAI-I* plasminogen activator inhibitor 1 antigen

In all groups, bacterial infusion caused a strong increase in circulating inflammatory cytokines. TNF-α and IL-10 peaked, prior to treatment, 2 h after the start of bacterial infusion without differences among groups. IL-6 significantly changed over time (*p* < 0.001). The highest concentration of IL-6 was reached 10 h after termination of the bacterial infusion (TP 12) in the CO group. In contrast, maximum IL-6 concentrations were measured at TP 8 in the LD-SATI group and at TP 6 in the HD-SATI group, respectively. SATI treatment in both arms significantly attenuated the magnitude of IL-6 release compared to CO (both *p* < 0.0001) (Fig. 5a-c).

Temperature was not different between the groups (data not shown). Similarly, circulating LBP concentrations were not different between the groups over the observation period.

### Hemodynamic response to sepsis

MAP, CI, SVRI and PVRI significantly changed over time (all *p* < 0.03) (Table 1). Administration of SATI led to higher CI, and lower SVRI and PVRI. Base excess (BE) significantly decreased over the observation period in all groups (*p* < 0.0001). However, SATI administration resulted in significantly improved BE in both treatment arms compared to the CO group from TP 8 until the end of the experiment (*p* < 0.01). Noradrenaline was required to maintain MAP >70 mmHg in seven out of eight animals in the CO group, compared to three out of eight in the LD-SATI group and one out of six in the HD-SATI group (*p* < 0.03). Nevertheless, two baboons died in the LD-SATI group: one after 16 h and one after 19 h due to hemodynamic failure but not to bleeding complications.

**Table 1:** Haemodynamic variables in the control group and both SATI groups

		0 h	1 h	2 h	4 h	6 h	8 h	12 h	16 h	20 h	24 h	*P* value
HR bpm	CO	118 ± 22	126 ± 24	159 ± 20	158 ± 22	148 ± 20	144 ± 15	142 ± 16	146 ± 19	154 ± 23	155 ± 10	0.741*
	LD SATI	127 ± 9	137 ± 18	160 ± 11	157 ± 16	151 ± 16	154 ± 14	148 ± 10	150 ± 21	143 ± 17	147 ± 19	0.593^#^
	HD SATI	128. ±26	140 ± 20	158 ± 22	148 ± 20	144 ± 15	161 ± 16	155 ± 15	153 ± 19	157 ± 22	157 ± 22	0.263^§^
MAP mmHg	CO	114 ± 18	113 ± 19	103 ± 15	110 ± 12	125 ± 10	121 ± 22	115 ± 12	90 ± 20	74 ± 12	73 ± 6	0.591*
	LD SATI	122 ± 13	125 ± 11	102 ± 15	112 ± 12	114 ± 11	121 ± 14	116 ± 16	102 ± 25	97 ± 23	100 ± 13	0.984^#^
	HD SATI	114 ± 32	115 ± 14	98 ± 11	100 ± 13	112 ± 14	112 ± 18	110 ± 17	106 ± 20	91 ± 22	91 ± 22	0.534^§^
MPAP mmHg	CO	15 ± 3	15 ± 4	17 ± 3	17 ± 6	23 ± 5	23 ± 2	30 ± 6	34 ± 6	27 ± 6	24 ± 6	0.993*
	LD SATI	14 ± 4	14 ± 3	15 ± 3	18 ± 4	20 ± 3	22 ± 4	32 ± 6	34 ± 6	29 ± 5	28 ± 5	0.687^#^
	HD SATI	15 ± 2	15 ± 2	16 ± 2	18 ± 3	21 ± 3	22 ± 4	25 ± 6	27 ± 6	25 ± 5	26 ± 5.	0.780^§^
PAOP mmHg	CO	6 ± 3	6 ± 2	6 ± 2.	6 ± 2	5 ± 3	6 ± 2	5 ± 2	6 ± 2	5 ± 2	7 ± 2	0.895*
	LD SATI	6 ± 2	7 ± 2	6 ± 1	5 ± 1	6 ± 1	6 ± 2	5 ± 1	6 ± 2	5 ± 2	5 ± 1	0.162^#^
	HD SATI	6 ± 2	6 ± 1	6 ± 2	4 ± 1	4 ± 1	5 ± 1	4 ± 1	5 ± 1	4 ± 1	4 ± 1	0.374^§^
SVRI dyn/sec/cm^-5^	CO	3477 ± 1190	2658 ± 763	2036 ± 418	3023 ± 1177	3987 ± 841	4797 ± 1618	4656 ± 1514	3080 ± 994	2246 ± 869.1	1817 7 ± 761	1.00*
	LD SATI	3445 ± 1198	2958 ± 885	2284 ± 717	2834 ± 619	2913 ± 612	3349 ± 673	3600 ± 826	3149 ± 761	2701 ± 600	2495 ± 589	0.318^#^
	HD SATI	2873 ± 860	2366 ± 418	1856 ± 270	2258 ± 313	2587 ± 437	2646 ± 484	2480 ± 547	2548 ± 734	2034 ± 518	2034 ± 518	0.368^§^
PVRI dyn/sec/cm^-5^	CO	273 ± 125	240 ± 119	207 ± 81	346 ± 294	610 ± 291	705 ± 235.1	1011 ± 323	989 ± 368	639 ± 234	445 ± 238	0.855*
	LD SATI	241 ± 125	178 ± 44	207 ± 50	322 ± 99	361 ± 73	449 ± 112.4	883 ± 342	1002 ± 501.9	717 ± 297	576 ± 168	0.324^#^
	HD SATI	236 ± 58	195 ± 35	204 ± 44	337 ± 77	403 ± 235	424 ± 111	470 ± 158	558 ± 189	511 ± 177	512 ± 177	0.657^§^
CI L/min/m^2^	CO	2.8 ± 1.1	3.5 ± 1.1	4.3 ± 1.3	3.1 ± 0.9	2.5 ± 0.5	2.1 ± 0.7	2.1 ± 0.7	2.5 ± 0.9	2.9 ± 1.1	3.5 ± 1.2	0.828*
	LD SATI	2.9 ± 0.8	3.6 ± 0.8	3.6 ± 0.8	3.1 ± 0.5	3.1 ± 0.5	2.9 ± 0.5	2.6 ± 0.5	2.6 ± 0.8	2.9 ± 0.8	3.2 ± 0.7	0.90^#^9
	HD SATI	3.1 ± 0.6	3.8 ± 0.6	4.1 ± 0.6	3.5 ± 0.7	3.5 ± 0.8	3.4 ± 0.8	3.5 ± 0.7	3.3 ± 0.5	3.6 ± 0.9	3.6 ± 0.9	0.622^§^
Base excess mmol/L	CO	4.1 ± 2.0	0.9 ± 2.6	1.4 ± 2.0	-0.9 ± 2.5	-0.9 ± 3.0	-2.0 ± 2.8	-7.4 ± 3.3	-9.3 ± 3.9	-12.8 ± 4.8	-13.9 ± 6.0	0.024*
	LD SATI	2,5 ± 4.3	0.8 ± 2.2	-0.9 ± 2.1	-1,5 ± 1.6	-2.1 ± 1.9	-2.3 ± 2.3	-3.8 ± 3.2	-7.4 ± 7.1	-7.3 ± 6.4	-3.7 ± 2.0	0.148^#^
	HD-SATI	2.8 ± 2.0	-1.0 ± 2.4	-0.7 ± 2.2	-1.0 ± 2.8	-2.6 ± 2.2	-1.8 ± 2.9	-4.3 ± 2.8	-4.1 ± 2.4	-6.1 ± 3.3	-6.1 ± 4.9	0.655^§^

Values are presented as mean and SD. *HR* heart rate, *MAP* mean artery pressure, *MAPA* mean pulmonary artery pressure, *PACP* pulmonary artery occlusion pressure, *SVRI* systemic vascular resistance index; pulmonary vascular resistance index; *CI* cardiac index, *CO* control group, *HD-SATI*, high-dose activated factor II and activated factor X inhibitor group; LD-SATI, high-dose activated factor II and activated factor X inhibitor group. *P* values are given for between-subjects analysis: *control vs LD-SATI; ^#^control vs HD-SATI; ^§^LD-SATI vs HD-SATI

### Blood cell count after *E. coli* infusion

All animals developed anemia and neutropenia after infusion of *E. coli* (Table 2). No significant differences were observed between the CO and the SATI groups.

**Table 2:** Blood cell count

		0 h	1 h	2 h	4 h	6 h	8 h	12 h	16 h	20 h	24 h	*P* value
Erythrocytes Mio/μL	CO	5.8 ± 0.4	5.8 ± 0.4	5.7 ± 0.5	5.9 ± 0.5	6.1 ± 0.6	6.2 ± 0.6	6.1 ± 0.7	5.7 ± 0.7	5.1 ± 0.8	4.5 ± 0.8	0.716*
	LD SATI	5.8 ± 0.5	5.7 ± 0.4	5.7 ± 0.5	5.6 ± 0.6	5.7 ± 0.5	5.8 ± 0.6	5.9 ± 0.6	5.3 ± 0.6	4.9 ± 0.8	4.7 ± 0.5	0.511^#^
	HD SATI	5.8 ± 0.4	5.8 ± 0.5	5.7 ± 0.6	5.8 ± 0.7	6.0 ± 0.8	6.0 ± 0.8	5.9 ± 0.8	5.8 ± 0.8	5.5 ± 0.8	5.1 ± 0.8	0.194^§^
Hct %	CO	44.7 ± 2.3	44.7 ± 2.1	44.7 ± 2.3	45.2 ± 3.1	46.6 ± 3.7	47.5 ± 3.8	46.7 ± 4.6	43.4 ± 5.2	39.8 ± 6.1	35.6 ± 6.3	0.327*
	LD SATI	43.9 ± 2.9	43.0 ± 2.0	43.0 ± 4.0	42.4 ± 2.9	42.9 ± 2.9	43.4 ± 3.3	42.2 ± 3.0	40.0 ± 3.5	36.8 ± 4.7	35.7 ± 3.4	0.980^#^
	HD SATI	43.9 ± 2.9	43.7 ± 3.3	45.2 ± 3.1	43.8 ± 5.3	45.0 ± 5.9	45.4 ± 6.2	44.4 ± 6.1	43.6 ± 5.9	41.2 ± 6.2	38.8 ± 1.3	0.299^§^
Hb g/dL	CO	14.6 ± 0.9	14.8 ± 1.1	14.6 ± 1.3	14.9 ± 0.9	15.5 ± 1.4	15.7 ± 1.5	15.6 ± 1.8	14.5 ± 2.0	13.3 ± 2.1	11.8 ± 2.1	0.460*
	LD SATI	14.7 ± 1.0	14.5 ± 0.7	14.4 ± 0.9	14.3 ± 1.1	14.4 ± 0.9	14.6 ± 1.0	14.3 ± 1.1	13.3 ± 1.2	12.2 ± 1.8	12.0 ± 1.2	0.918^#^
	HD SATI	14.2 ± 1.2	14.3 ± 1.2	14.0 ± 1.4	14.3 ± 1.8	14.6 ± 2.1	14.6 ± 2.3	14.3 ± 2.2	14.1 ± 2.0	13.2 ± 2.0	12.2 ± 2.0	0.321^§^
WBC G/L	CO	8,994 ± 2.951	4,684 ± 2.089	1,323 ± 0.375	1,463 ± 791	1,750 ± 1603	2,454 ± 3,026	1,090 ± 493	5,689 ± 7,504	7,244 ± 9,355	6,935 ± 9,047	0.988*
	LD SATI	7,413 ± 3.200	5,141 ± 1.831	2,099 ± 1.773	1,645 ± 681	1,619 ± 1.066	1,864 ± 1,757	3,424 ± 3,471	4,271 ± 4,009	5,391 ± 3,863	7,523 ± 4,379	0.547^#^
	HD SATI	8,250 ± 3.47	3,417 ± 0.80	1,250 ± 0,476	1,233 ± 423	867 ± 186	833 ± 151	1,383 ± 256	1,833 ± 459	2,350 ± 764	3,267 ± 1,515	0.493^§^

Values are presented as mean and SD. *Hct* hematocrit, *Hb* hemoglobin, *WBC* white blood cell count, *CO* control; HD-SATI; high-dose activated factor II and activated factor X inhibitor-group; LD-SATI; high-dose activated factor II and activated factor X inhibitor-group. *P* values are given for between-subjects analysis: *control vs LD-SATI; ^#^control vs HD-SATI; ^§^LD-SATI vs HD-SATI

## Discussion

This is the first study investigating the effect of dual inhibition of thrombin and activated FX in a non-human primate model of severe Gram-negative sepsis. Post-treatment with SATI starting 2 h and 15 minutes after the initiation of sepsis blocked thrombin and factor X-mediated activation of coagulation and maintained natural inhibitors such as ATIII and APC. Moreover, profibrinolytic pathways were preserved in particular in those animals that received HD-SATI. Most importantly, treatment with SATI, regardless of the dose, strongly attenuated sepsis-induced organ injury reflected by the reduced release of organ-specific enzymes and even prevented anuria, which developed after 8 h. The results of the current study indicate that dual inhibition of thrombin and FXa by SATI is capable of attenuating the severity of DIC and counteracts sepsis-induced organ dysfunction. Furthermore, SATI abolished the late inflammatory response as indicated by diminished release of circulating IL-6.

We used a baboon model, which closely recapitulates early events in human sepsis such as hyperdynamic response in the acute phase, even with adequate fluid resuscitation [24–26]. The bacterial challenge was severe enough to provoke a massive inflammatory response, strong activation of coagulation and sustained organ damage, but allowed the majority of animals to survive for 24 h; only two baboons died before completion of the experiment. At the end of the bacterial infusion, all groups had a similar septic phenotype: high inflammatory response, hemodynamic instability and strong upregulation of procoagulant pathways.

The protracted infusion of *E.coli* strongly increased thrombin generation as indicated by a very high concentration of TAT complexes in the CO group. ATIII binds thrombin in a 1:1 ratio leading to formation of TAT [27]. In contrast, SATI treatment robustly reduced active thrombin binding to ATIII, resulting in lower TAT via two modalities. On one hand SATI blocks the active site of thrombin and consequently inhibits binding to ATIII. On the other hand it can be partly related to the reduced thrombin production by SATI via blockade of Xa activity (dual-action SATI concept). Thus, ATIII decreased significantly more in the CO group compared to both SATI groups due to higher consumption by free thrombin, consequently leading to stronger elevation in TAT in the CO group. Other inhibitors such as APC and TFPI increased to a greater extent in the CO group compared to the SATI groups up to the end of the experiment. As mentioned, this effect could be explained by the fact that SATI blocks the active thrombin site, consequently hindering binding on TM. Thus, activation of protein C is downregulated by SATI, which led to a higher concentration of APC in the CO group.

In the present study, the marked increase in PAI-1 was significantly ameliorated by HD-SATI. High concentrations of inflammatory cytokines such as TNF-α and IL-1 provoke release of PAI-1 into the bloodstream, causing sustained inhibition of thrombolysis [28]. In experimental and clinical studies, high PAI-1 has been strongly associated with poor outcome in severe sepsis [29, 30].

Additionally, we observed strong upregulation of TAFI in the CO group, which further hinders lysis. This effect was in part counteracted by high APC in the CO group, which in turn inactivates PAI-1. D-dimer elevation in the CO group could result from a sustained increase in fibrin formation (related to high thrombin availability) rather than being per se a marker of upregulated lysis.

Interestingly thrombocytopenia was not different between the groups. This is a remarkable finding in the light that SATI robustly abolished thrombin generation as indicated by significantly lower TAT. A possible explanation for this observation is that this severe life-threatening sepsis resulted in sustained upregulation of platelet action, which in turn caused pronounced thrombocytopenia independent of therapeutic intervention.

Hemodynamic instability and organ dysfunction is a common issue in severe sepsis and septic shock [10]. Intravascular fibrin formation results in limited nutrient delivery to vital organs and consecutive end-organ damage. In particular, tissues with high oxygen demand such as the kidneys are susceptible to compromised end-organ perfusion [31]. The BE was significantly lower in the CO group compared to both SATI treatment arms, which might suggest improved microvascular blood flow. While all CO animals stopped urine production after the first 8 h, remarkably, diuresis in both SATI groups was maintained until the end of the experiment. The sepsis-induced organ dysfunction in our study closely meets the most recent definition of sepsis [32]. Over the study course, the lung, liver, kidney and pancreas function deteriorated. The life-threatening organ failure observed in the CO group was significantly improved in baboons treated with SATI. In particular, HD-SATI preserved organ function. It is important to note that, in both SATI groups compared to the CO, fewer catecholamines were necessary to maintain MAP >70 mmHg.

Several randomized controlled studies have been conducted using natural anticoagulants such as ATIII [13, 14], recombinant human APC [12, 16], recombinant human TFPI [12, 15] or recombinant human TM [33] in patients with sepsis. Those drugs reduced the hemostatic abnormalities in sepsis but did not improve survival [34]. However, on post-hoc subgroup analysis restricted to those patients with overt DIC, there were survival benefits in both the Kybersept study and the PROWESS trial [17, 18]. This strongly implies the need for a more homogenous stratification of patients with sepsis into those with overt DIC and those with sepsis but non-overt DIC. Patients with established DIC could benefit from treatment with anticoagulants such as ATIII or APC. Our data provide evidence that dual inhibition of both thrombin and FXa robustly ameliorates organ dysfunction, most likely by improving microvascular nutrient blood flow based on diminished intravascular fibrin formation.

Another important finding of the current study is that SATI treatment was highly effective in preventing the progression of DIC but there were no signs that it provoked any severe bleeding events, even in the HD-SATI group. This is in strong contrast to studies testing therapeutic administration of ATIII and APC, which led to life-threatening bleeding complications [12, 13]. Giardino et al. confirmed that compared to selective inhibition alone, dual inhibition of thrombin and FXa exerts a superior antithrombotic efficacy with a tendency toward diminished bleeding [22].

The bacterial infusion provoked a potent release of initial inflammatory cytokines prior to the onset of treatment. Thus, SATI could not have meaningful effect on the time course and magnitude of TNF-α and IL-10, which peaked at the end of the bacterial challenge, long before the start of treatment. IL-6 release occurs later than that of TNF-α and IL-10; however, the pattern of production is somewhat different in various models [35]. Both SATI groups were effective in attenuating late IL-6 release in the bloodstream. IL-6 has been shown to be, at least in part, under the control of the initial TNF-α release [36]. Considering that TNF-α release prior to the SATI treatment was not affected, we assume that SATI per se has a direct effect on IL-6 neo-synthesis. Alternatively, the observed effect could be a consequence of diminished availability of thrombin, which has strong pro-inflammatory properties [4].

### Limitation

Due to the limited observation period, our study does not provide any relevant outcome information on the efficacy of SATI at later stages. Early empiric antibiotic treatment is an essential part of initial treatment of patients with sepsis [10]. In the current short-term study, we did not use any additional antibiotic therapy, which could potentially have influence on the time course of sepsis-related organ damage. However, SATI treatment according to our protocol was effective in improving sepsis-related organ injury.

The duration of the study was too short to completely rule out substantial bleeding complications at a later stage. However, even in the HD-SATI group relevant bleeding episodes were not observed, including external bleeding and mucosa or gastrointestinal bleeding, which would have been apparent after harvesting the organs post mortem. Two animals in the LD-SATI group died due to hemodynamic decompensation. However, it has to be noted that the study was not powered to show any survival differences between groups.

## Conclusion

SATI treatment diminished thrombin formation and preserved anticoagulant and fibrinolytic pathways. This effect coincided with an effective and robust reduction in severe sepsis-induced organ injury in both SATI treatment groups. Moreover, dual inhibition of thrombin and aFX with SATI strongly ameliorated late inflammation in baboons with sepsis.

## Key messages

- Bacterial invasion prompts a compelling upregulation of the coagulation system and inhibits anticoagulant and fibrinolytic pathways, which results in widespread microvascular fibrin deposition
- Dual inhibition of activated FII and activated FX (SATI) diminishes thrombin formation and preserves anticoagulant and fibrinolytic pathways
- SATI administration strongly ameliorates IL-6 release in severe sepsis
- SATI robustly attenuates sepsis-induced organ damage and protects organ function

## Abbreviations

ALT: Alanine-aminotransferase
APC: Activated protein C
AST: Aspartate-aminotransferase
AT III: Antithrombin III
BE: Base excess
BW: Body weight
CI: Cardiac index
CK: Creatine kinase
CO: Control group
CVP: Central venous pressure
DIC: Disseminated intravascular coagulation
ELISA: Enzyme-linked immunosorbent assay
FiO_2_/paO_2_: Oxygenation ratio
FIX: Factor IX
FVII: Factor VII
FX: Factor X
Hb: Hemoglobin
Hct: Hematocrit
HD-SATI: High dose short acting thrombosis inhibitor group
IL-10: Interleukin-10
IL-6: Interleukin-6
LBP: Lipopolysaccharide binding protein
LDH: Lactate-dehydrogenase
LD-SATI: Low dose short acting thrombosis inhibitor group
MAP: Mean arterial pressure
PAI-1: Plasminogen activator inhibitor 1 antigen
PAOP: Pulmonary artery occlusion pressure
PVRI: Pulmonary vascular resistance index
SATI: Short-acting thrombosis inhibitor
SVRI: Systemic vascular resistance index
TAFI: Thrombin activated fibrinolysis inhibitor antigen
TAT: Thrombin-antithrombin-complexes
TF: Tissue factor
TFPI: Tissue factor pathway inhibitor
TM: Thrombomodulin
TNF-α: Tumor necrosis factor-α
TP: Time point
